# Supplementary figures and images for: Analysis of Bacterial and Metabolic Diversity of Pickles in Different Karst Regions of Guizhou, China
Source: Foods. 2025 Apr 11;14(8):1324. doi: 10.3390/foods14081324 (PMC12027304; doi:10.3390/foods14081324)

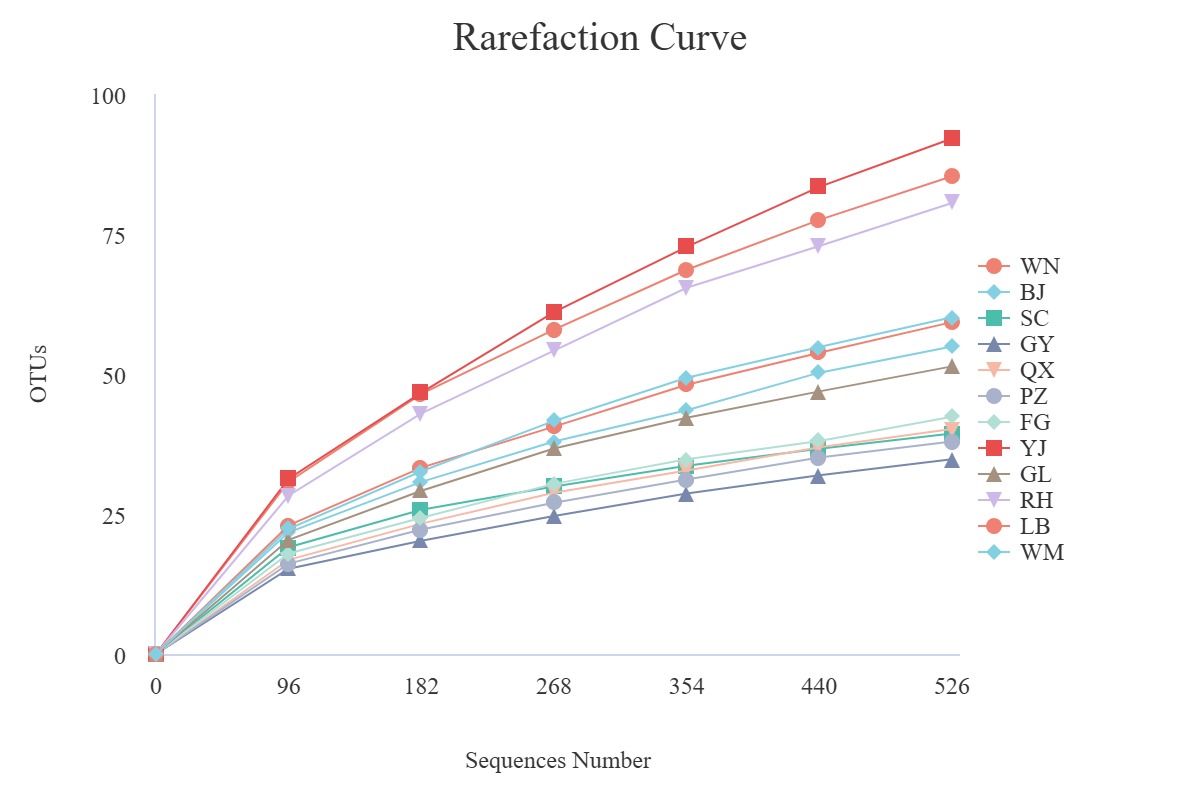

Supplement: Supplementary file 1 [file foods-14-01324-s001.zip › Figure S1 rarefaction-curve.jpeg]

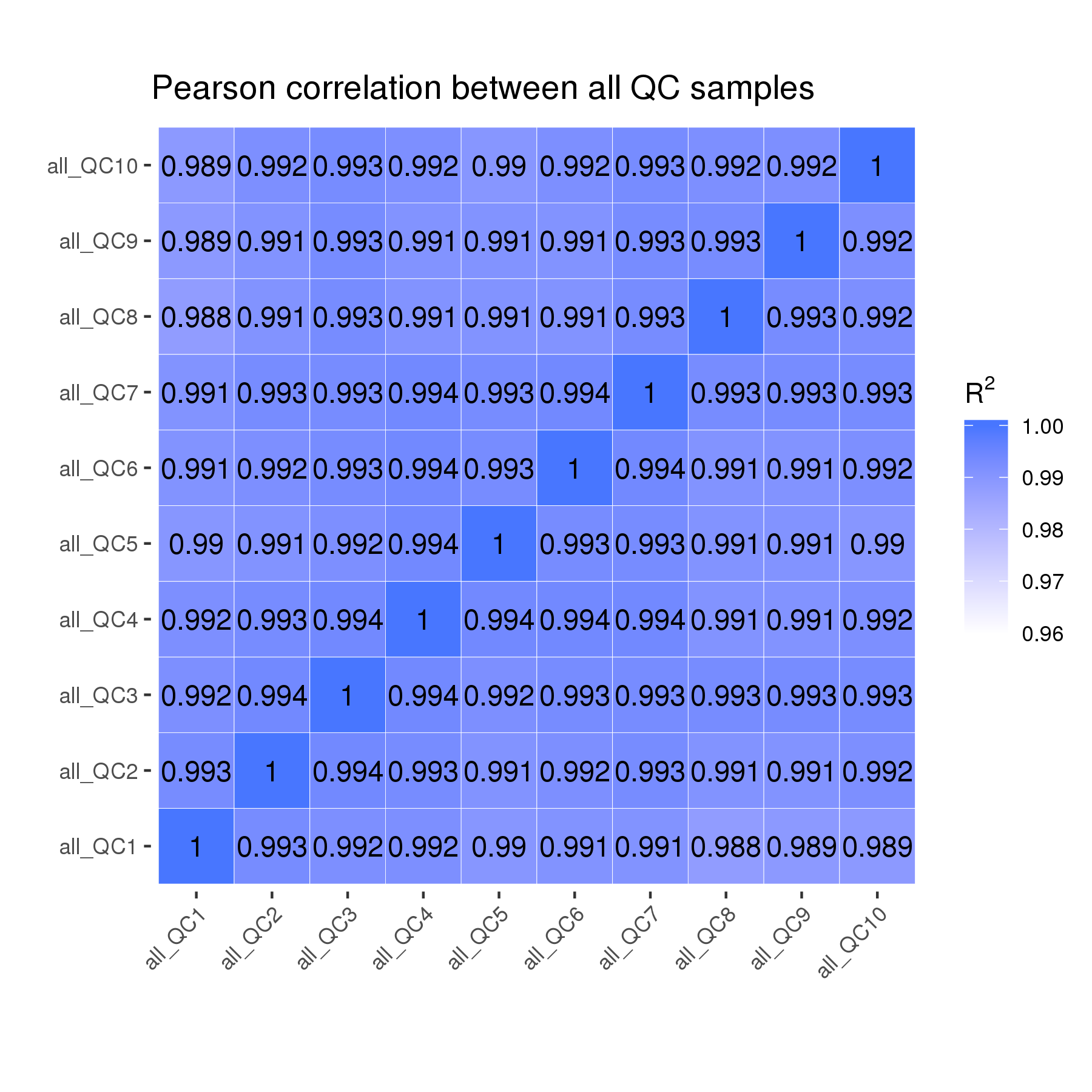

Supplement: Supplementary file 1 [file foods-14-01324-s001.zip › Figure S2 QC .png]
